# Supplementary material for: Taking the opportunity of COVID testing to screen vulnerable populations for hepatitis B, hepatitis C, syphilis, and human immunodeficiency virus in Central Brazil
Source: PLoS One. 2025 Jul 11;20(7):e0325859. doi: 10.1371/journal.pone.0325859 (PMC12250198; doi:10.1371/journal.pone.0325859)
Supplement: S1 Table — (DOCX) [file pone.0325859.s001.docx]

**S1 Table. Characteristics of vulnerable people, according to the group in Goiânia, Central Brazil**

| Variable | waste recycler picker | % | Immigrant/  refugee | % | Homeless | % | LGBT | % |
| --- | --- | --- | --- | --- | --- | --- | --- | --- |
|  | n/Total* |  | n/Total |  | n/Total |  | n/Total |  |
| **Male gender** | 138/283 | 48.8 | 73/165 | 44.2 | 92/140 | 65.7 | 59/99 | 59.6 |
| **Physical violence** | 5/283 | 1.8 | 5/165 | 3.0 | 21/140 | 15.0 | **10/99** | **10.1** |
| **Coercive sex** | - |  | - |  | 2/140 | 1.4 | **1/99** | **1.0** |
| **Transactional sex** | 21/279 | 7.5 | 1/169 | 0.6 | 21/132 | 15.9 | 28/97 | 28.9 |
| **Non-condom use in the last sexual intercourse** | 165/276 | 59.8 | 116/155 | 74.8 | 66/123 | 53.7 | 45/98 | 45.9 |
| **STI^a^ report** | 43/277 | 15.5 | 9/160 | 5.6 | 38/137 | 27.7 | 40/99 | 40.4 |
| **Anal sex** | 100/275 | 36.4 | 28/155 | 18.1 | 55/133 | 41.4 | 79/99 | 79.8 |
| **Daily alcohol consumption** | 13/283 | 4.6 | 2/165 | 1.2 | 14/140 | 10.0 | 5/99 | 5.1 |
| **Illicit drug use** | 85/283 | 30.0 | 12/163 | 7.4 | 66/138 | 47.8 | 57/99 | 57.6 |
| **Previous arrest** | 41/281 | 14.6 | 8/158 | 5.1 | 45/138 | 32.6 | 12/98 | 12.2 |
| **Continuous variables** | **Median** | **IQR^c^** | **Median** | **IQR^c^** | **Median** | **IQR^c^** | **Median** | **IQR^c^** |
| **Number of partners (last month)** | 1 | 0 | 1 | 0 | 1 | 2 | 1 | 2 |
| **Age** | 37 | 20 | 30 | 13 | 36 | 25 | 30 | 16 |
| **Monthly income (R$)^b^** | 1,500 | 1,155 | 1,200 | 325 | 1,150 | 600 | 2,000 | 1,950 |
| **Time of scholarly** | 9 | 7 | 12 | 4 | 9 | 5 | 12 | 4 |

*Only valid data.

^a^STI: sexually transmitted infection

^b^ IQR: interquartile range

^c^ R$ 5,64 was equivalent to US$
