# Supplementary material for: Taking the opportunity of COVID testing to screen vulnerable populations for hepatitis B, hepatitis C, syphilis, and human immunodeficiency virus in Central Brazil
Source: PLoS One. 2025 Jul 11;20(7):e0325859. doi: 10.1371/journal.pone.0325859 (PMC12250198; doi:10.1371/journal.pone.0325859)
Supplement: S2 Table — (DOCX) [file pone.0325859.s002.docx]

**S2 Table.** Bivariate analysis of potential variables associated with anti-HCV among vulnerable people in Goiânia, Central Brazil

| **Variable** | **Neg. (%)** | **Pos.(%)** | ***p-value*** |
| --- | --- | --- | --- |
| **Waste recycle pickers** |  |  |  |
| No | 336 (97.7) | 8 (2.3) |  |
| Yes | 279 (98.6) | 4 (1.4) | 0.561 |
| **Immigrants/Refugees** |  |  |  |
| No | 450 (97.4) | 12 (2.6) |  |
| Yes | 165 (100) | 0 (0) | **0.043** |
| **Homeless** |  |  |  |
| No | 483 (99.2) | 4 (0.8) |  |
| Yes | 132 (94.3) | 8 (5.7) | **0.001** |
| **LGBT** |  |  |  |
| No | 517 (97.9) | 11 (2.1) |  |
| Yes | 98 (99) | 1 (1.0) | 0.702 |
| **Gender** |  |  |  |
| Male | 325 (97.9) | 7 (2.1) |  |
| Female | 290 (98.3) | 5 (1.7) | 0.706 |
| **White Color** |  |  |  |
| Yes | 108 (98.2) | 2 (1.8) |  |
| No | 506 (98.1) | 10 (1.9) | 1.000 |
| **Physical violence** |  |  |  |
| No | 585 (98.2) | 11 (1.8) |  |
| Yes | 30 (96.8) | 1 (3.2) | 0.459 |
| **Anal sex** |  |  |  |
| No | 360 (98.4) | 6 (1.6) |  |
| Yes | 206 (86.6) | 32 (13.4) | 0.071 |
| **Condom use (last sexual intercourse)** |  |  |  |
| Yes | 230 (96.6) | 8 (3.4) |  |
| No | 353 (98.9) | 4 (1.1) | 0.075 |
| **STI report** |  |  |  |
| No | 491 (99.2) | 4 (0.8) |  |
| Yes | 112 (94.1) | 7 (5.9) | **0.001** |
| **Transactional** |  |  |  |
| No | 536 (98) | 11 (2.0) |  |
| Yes | 62 (82.5) | 1 (1.6) | 1.000 |
| **Illicit drug use** |  |  |  |
| No | 421 (99.3) | 3 (0.7) |  |
| Yes | 190 (95.5) | 9 (4.5) | **0.003** |
| **Daily alcohol consumption** |  |  |  |
| No | 587 (98.2) | 11 (1.8) |  |
| Yes | 28 (96.6) | 1 (3.4) | 0.436 |
| **Previous arrest** |  |  |  |
| No | 511 (98.8) | 6 (1.2) |  |
| Yes | 92 (93.9) | 6 (6.1) | **0.006** |
| **Awareness of HIV diagnosis** |  |  |  |
| **No** | 591 (98.7) | 8 (1.3) |  |
| **Yes** | 24 (85.7) | 4 (14.3) | **0.001** |
| **Number of sexual partners in the last month (Median; IQR)** | 1 (0) | 1 (2) | 0.236 |
| **Age (Median; IQR)** | 33 (20) | 50 (11.3) | **< 0.001** |
| **Schooling in years (Median; IQR)** | 10 (6) | 7 (6) | 0.04 |
| **Monthly income (R$)(Median; IQR)** | 1,200 (976) | 1,056 (1,090) | 0.027 |
