# Supplementary material for: Taking the opportunity of COVID testing to screen vulnerable populations for hepatitis B, hepatitis C, syphilis, and human immunodeficiency virus in Central Brazil
Source: PLoS One. 2025 Jul 11;20(7):e0325859. doi: 10.1371/journal.pone.0325859 (PMC12250198; doi:10.1371/journal.pone.0325859)
Supplement: S3 Table — (DOCX) [file pone.0325859.s003.docx]

**S3 Table.** Bivariate analysis of potential variables associated with HBV among vulnerable people in Goiânia, Central Brazil

| **Variable** | **Neg. (%)** | **Pos.(%)** | ***p-value*** |
| --- | --- | --- | --- |
| **Waste recycle pickers** |  |  |  |
| No | 135 (67.8) | 64 (32.2) |  |
| Yes | 144 (79.6) | 37 (20.4) | **0.010** |
| **Immigrants/Refugees** |  |  |  |
| No | 226 (77.1) | 67 (22.9) |  |
| Yes | 53 (60.9) | 34 (39.1) | **0.003** |
| **Homeless** |  |  |  |
| No | 209 (73.6) | 75 (26.4) |  |
| Yes | 70 (72.9) | 26 (27.1) | 0.897 |
| **LGBT** |  |  |  |
| No | 244 (74.8) | 82 (25.2) |  |
| Yes | 35 (64.8) | 19 (35.2) | 0.122 |
| **Gender** |  |  |  |
| Male | 155 (74.5) | 53 (25.5) |  |
| Female | 124 (72.1) | 48 (27.9) | 0.594 |
| **White color** |  |  |  |
| Yes | 51(773) | 15 (22.7) |  |
| No | 228 (77.8) | 85 (27.2) | 0.458 |
| **Physical violence** |  |  |  |
| No | 266 (73.5) | 96 (26.5) |  |
| Yes | 13 (72.2) | 5 (27.8) | 0.906 |
| **Anal sex** |  |  |  |
| No | 160 (71.7) | 63 (28.3) |  |
| Yes | 105 (75.5) | 34 (24.5) | 0.428 |
| **Condom use (last sexual intercourse)** |  |  |  |
| Yes | 99 (68.8) | 45 (31.3) |  |
| No | 159 (75.4) | 52 (24.6) | 0.170 |
| **STI report** |  |  |  |
| No | 229 (76.1) | 72 (23.9) |  |
| Yes | 41 (60.3) | 27 (39.7) | **0.008** |
| **Transactional sex** |  |  |  |
| No | 244 (74.2) | 85 (25.8) |  |
| Yes | 24 (61.5) | 15 (38.5) | 0.094 |
| **Illicit drug use** |  |  |  |
| No | 179 (70.5) | 75 (29.5) |  |
| Yes | 97 (78.9) | 26 (21.1) | 0.085 |
| **Daily alcohol consumption** |  |  |  |
| No | 262 (73.0) | 97 (27.0) |  |
| Yes | 17 (81.0) | 4 (19.0) | 0.612 |
| **Previous arrest** |  |  |  |
| No | 227 (74.4) | 78 (25.6) |  |
| Yes | 47 (70.1) | 20 (29.9) | 0.472 |
| **Aware of HIV diagnosis** |  |  |  |
| No | 271 (74.9) | 91 (25.1) |  |
| Yes | 8 (44.4) | 10 (55.6) | **0.004** |
| **Number of sexual partners in the last month (Median; IQR)** | 1 (0) | 1 (1) | 0.216 |
| **Age (Median; IQR)** | 33 (19) | 42 (26) | **0.007** |
| **Schooling in years (Median; IQR)** | 10 (5) | 9 (7) | 0.422 |
| **Monthly income (R$)(Median; IQR)** | 1,200 (1,010) | 1.200 (600) | 0.422 |
