# Supplementary material for: Taking the opportunity of COVID testing to screen vulnerable populations for hepatitis B, hepatitis C, syphilis, and human immunodeficiency virus in Central Brazil
Source: PLoS One. 2025 Jul 11;20(7):e0325859. doi: 10.1371/journal.pone.0325859 (PMC12250198; doi:10.1371/journal.pone.0325859)
Supplement: S4 Table — (DOCX) [file pone.0325859.s004.docx]

**S4 Table.** Bivariate analysis of potential variables associated with syphilis among vulnerable people in Goiânia, Central Brazil

| **Variable** | **Neg. (%)** | **Pos.(%)** | ***p-value*** |
| --- | --- | --- | --- |
| **Waste recycle pickers** |  |  |  |
| No | 268 (77.9) | 76 (22.1) |  |
| Yes | 251 (88.7) | 32 (11.3) | **< 0.001** |
| **Immigrants/Refugees** |  |  |  |
| No | 367 (79.4) | 95 (20.6) |  |
| Yes | 152 (92.1) | 13 (7.9) | **< 0.001** |
| **Homeless** |  |  |  |
| No | 418 (85.8) | 69 (14.2) |  |
| Yes | 101 (72.1) | 39 (27.9) | **< 0.001** |
| **LGBT** |  |  |  |
| No | 457 (86.6) | 71 (13.4) |  |
| Yes | 62 (62.6) | 37 (37.4) | **< 0.001** |
| **Gender** |  |  |  |
| Male | 270 (81.3) | 62 (18.7) |  |
| Female | 249 (84.4) | 46 (15.6) | 0.308 |
| **White Color** |  |  |  |
| Yes | 93 (84.5) | 17 (15.5) |  |
| No | 425 (82.5) | 91 (17.6) | 0.583 |
| **Physical violence** |  |  |  |
| No | 499 (83.7) | 97 (16.3) |  |
| Yes | 20 (64.5) | 11 (35.5) | **0.006** |
| **Anal sex** |  |  |  |
| No | 325 (88.8) | 41 (11.2) |  |
| Yes | 174 (73.1) | 64 (26.9) | **< 0.001** |
| **Condom use (last sexual intercourse)** |  |  |  |
| Yes | 179 (75.2) | 59 (24.8) |  |
| No | 311 (87.1) | 46 (12.6) | **< 0.001** |
| **STI report** |  |  |  |
| No | 447 (90.3) | 48 (9.7) |  |
| Yes | 61 (51.3) | 58 (48.7) | **< 0.001** |
| **Transactional sex** |  |  |  |
| No | 80 (14.6) | 82/323 |  |
| Yes | 39 (61.9) | 24 (38.1) | **< 0.001** |
| **Illicit drug use** |  |  |  |
| No | 367 (86.6) | 57 (13.4) |  |
| Yes | 148 (74.4) | 51 (25.6) | **<0.001** |
| **Daily alcohol consumption** |  |  |  |
| No | 499 (83.4) | 99 (16.6) |  |
| Yes | 20 (69) | 9 (31) | **0.044** |
| **Previous arrest** |  |  |  |
| No | 428 (84.7) | 79 (15.3) |  |
| Yes | 71 (72.4) | 27 (27.6) | **0.003** |
| **Aware of HIV diagnosis** |  |  |  |
| No | 506 (84.5) | 93 (15.5) |  |
| Yes | 13 (46.4) | 15 (53.6) | **<0.001** |
| **Number of sexual partners** **in the last month (Median; IQR)** | 1 (0) | 1 (2) | 0.150 |
| **Age (Median; IQR)** | 33 (20) | 38 (21.8) | **0.003** |
| **Schooling in years (Median; IQR)** | 10 (6) | 10 (6) | 0.591 |
| **Monthly income (R$)(Median; IQR)** | 1,200 (976) | 1,200 (1,000) | 0.722 |
