# Supplementary material for: Taking the opportunity of COVID testing to screen vulnerable populations for hepatitis B, hepatitis C, syphilis, and human immunodeficiency virus in Central Brazil
Source: PLoS One. 2025 Jul 11;20(7):e0325859. doi: 10.1371/journal.pone.0325859 (PMC12250198; doi:10.1371/journal.pone.0325859)
Supplement: S5 Table — (DOCX) [file pone.0325859.s005.docx]

**S5 Table.** Bivariate analysis of potential variables associated with HIV among vulnerable people in Goiânia, Central Brazil

| **Variable** | **Neg. (%)** | **Pos.(%)** | ***p-value*** | |
| --- | --- | --- | --- | --- |
| **Waste recycle pickers** |  |  |  |  |
| No | 308 (89.5) | 36 (10.5) |  |  |
| Yes | 281 (99.3) | 2 (0.7) | **< 0.001** |  |
| **Immigrants/Refugees** |  |  |  |  |
| No | 426 (92.2) | 36 (7.8) |  |  |
| Yes | 251 (98.8) | 2 (1.2) | **0.001** |  |
| **Homeless** |  |  |  |  |
| No | 465 (95.5) | 22 (4.5) |  |  |
| Yes | 124 (88.6) | 16 (11.4) | **0.004** |  |
| **LGBT** |  |  |  |  |
| No | 513 (97.2) | 15 (2.8) |  |  |
| Yes | 76 (76.8) | 23 (23.2) | **< 0.001** |  |
| **Gender** |  |  |  |  |
| Male | 397 (92.5) | 25 (7.5) |  |  |
| Female | 282 (95.6) | 13 (4.4) | 0.102 |  |
| **White Color** |  |  |  |  |
| Yes | 101 (91.8) | 19 (8.2) |  |  |
| No | 487 (94.4) | 29 (5.6) | 0.307 |  |
| **Anal sex** |  |  |  |  |
| No | 360 (98.4) | 6 (1.6) |  |  |
| Yes | 206 (86.6) | 32 (13.4) | **< 0.001** |  |
| **Condom use (last sexual intercourse)** |  |  |  |  |
| Yes | 211 (88.7) | 27 (11.3) |  |  |
| No | 346 (96.9) | 11 (3.1) | **< 0.001** |  |
| **STI report** |  |  |  |  |
| No | 490 (99) | 5 (1.0) |  |  |
| Yes | 86 (72.3) | 33 (27.7) | **< 0.001** |  |
| **Transactional sex** |  |  |  |  |
| No | 520 (95.1) | 27 (4.9) |  |  |
| Yes | 52 (82.5) | 11 (17.5) | **< 0.001** |  |
| **Illicit drug use** |  |  |  |  |
| No | 412 (97.2) | 12 (2.8) |  |  |
| Yes | 173 (86.9) | 26 (13.1) | **< 0.001** |  |
| **Alcohol daily consumption** |  |  |  |  |
| No | 561 (93.8) | 37 (6.2) |  |  |
| Yes | 28 (96.6) | 1 (3.4) | 1.000 |  |
| **Previous arrest** |  |  |  |  |
| No | 493 (95.4) | 24 (4.6) |  |  |
| Yes | 85 (86.7) | 13 (13.3) | **0.001** |  |
| **Number of sexual partners in the last month (Median; IQR)** | 1 (0) | 1 (2) | 0.532 |  |
| **Age (Median; IQR)** | 34 (20) | 35 (24) | 0.212 |  |
| **Schooling in years (Median; IQR)** | 10 (6) | 12 (6) | 0.201 |  |
| **Monthly income (R$)(Median; IQR)** | 1,200 (1,000) | 1,200 (780) | 0.674 |  |
