# Supplementary material for: Taking the opportunity of COVID testing to screen vulnerable populations for hepatitis B, hepatitis C, syphilis, and human immunodeficiency virus in Central Brazil
Source: PLoS One. 2025 Jul 11;20(7):e0325859. doi: 10.1371/journal.pone.0325859 (PMC12250198; doi:10.1371/journal.pone.0325859)
Supplement: Attachment — Submitted filename: PONE-D-24-55277 JBO.pdf [file pone.0325859.s006.pdf]

PLOS ONE

Screening vulnerable populations for hepatitis B, hepatitis C, syphilis, and human immunodeficiency virus during the coronavirus disease 2019 pandemic

--Manuscript Draft--

Manuscript Number:	PONE-D-24-55277
Article Type:	Research Article
Full Title:	Screening vulnerable populations for hepatitis B, hepatitis C, syphilis, and human immunodeficiency virus during the coronavirus disease 2019 pandemic
Short Title:	Hepatitis B, hepatitis C, syphilis, and human immunodeficiency among vulnerable people
Corresponding Author:	Sheila Araujo Teles, PhD<br>Universidade Federal de Goiás<br>Goiania, Goias BRAZIL
Keywords:	HIV; Syphilis; hepatitis; Vulnerable Populations; COVID-19
Abstract:	During the coronavirus disease 2019 pandemic, we investigated the prevalence of hepatitis B virus (HBV), hepatitis C virus (HCV), syphilis, and human immunodeficiency virus (HIV) among socio-economically vulnerable populations in Goiania, Central Brazil. From August 1st, 2020, to April 28th, 2021, a total of 627 individuals, including lesbian, gay, bisexual, and transgender (LGBT) people, homeless individuals, waste recyclable collectors, and immigrants/refugees, were tested for these infections. Multiple Poisson regression models were used to analyze the factors associated with each outcome. HBV markers were found in 16.1% of participants, antibodies against HCV (anti-HCV) in 1.9%, syphilis in 17.2%, and antibodies against HIV (anti-HIV) in 6.1%. Among these, 32 individuals exhibited serological evidence of active syphilis, whereas HBV deoxyribonucleic acid and HCV ribonucleic acid were detected in six and two individuals, respectively. All affected individuals were referred to health services for appropriate care. Increased age was statistically associated with HCV, HBV, and syphilis; being aware of an HIV diagnosis was linked to HCV and HBV; and identifying as LGBT and reporting a history of sexually transmitted infections was associated with syphilis and HIV. Drug use was associated with HCV and HIV, whereas being an immigrant/refugee or engaging in transactional sex was linked to HBV. Homelessness and reporting a higher number of sexual partners in the previous month were associated with syphilis. Conversely, condom use during the last sexual intercourse served as a protective factor. High prevalence rates of these infections were identified, with many participants showing evidence of active infections, increasing the risk of transmission. Specific risk behaviors were associated with each infection, emphasizing the need to tailor prevention strategies to address these behaviors effectively.
Order of Authors:	Gabriel Francisco da Silva Filho, Master
	Antoninho Barros Milhomem, Master
	Bruno Vinícius Diniz e Silva, Master
	Kamila Cardoso dos Santos, PhD
	Grazielle Rosa da Costa e Silva, Master
	Larissa Silva Magalhães, PhD
	Winny Éveny Alves Moura, Master
	Thaynara Lorrane Silva Martins, Master
	Wanessa de Oliveira Gonçalves, Bachelor
	Roxana Isabel Cardozo Gonzales, PhD
	Leonora Rezende Pacheco, PhD
	Regina Maria Bringel Martins, PhD
	Karlla Antonieta Amorim Caetano, PhD

Powered by Editorial Manager® and ProduXion Manager® from Aries Systems Corporation

	Megmar Aparecida dos Santos Carneiro, PhD
	Sheila Araujo Teles, PhD
Additional Information:	
Question	Response
Financial Disclosure<br><br>Enter a financial disclosure statement that describes the sources of funding for the work included in this submission. Review the submission guidelines for detailed requirements. View published research articles from PLOS ONE for specific examples.<br><br>This statement is required for submission and will appear in the published article if the submission is accepted. Please make sure it is accurate.<br><br>Funded studies<br>Enter a statement with the following details:<br>• Initials of the authors who received each award<br>• Grant numbers awarded to each author<br>• The full name of each funder<br>• URL of each funder website<br>• Did the sponsors or funders play any role in the study design, data collection and analysis, decision to publish, or preparation of the manuscript?<br><br>Did you receive funding for this work?	Yes
Please add funding details.<br>as follow-up to "Financial Disclosure<br><br>Enter a financial disclosure statement that describes the sources of funding for the work included in this submission. Review the submission guidelines for detailed requirements. View published research articles from PLOS ONE for specific examples.<br><br>This statement is required for submission and will appear in the published article if the submission is accepted. Please make	Fundação de Amparo à Pesquisa do Estado de Goiás - FAPEG, CHAMADA FAPEG No 05/2020

Powered by Editorial Manager® and ProduXion Manager® from Aries Systems Corporation

sure it is accurate.<br><br>Funded studies<br>Enter a statement with the following details:<br>• Initials of the authors who received each award<br>• Grant numbers awarded to each author<br>• The full name of each funder<br>• URL of each funder website<br>• Did the sponsors or funders play any role in the study design, data collection and analysis, decision to publish, or preparation of the manuscript?<br><br>Did you receive funding for this work?"	
Please select the country of your main research funder (please select carefully as in some cases this is used in fee calculation).<br>as follow-up to "Financial Disclosure<br><br>Enter a financial disclosure statement that describes the sources of funding for the work included in this submission. Review the submission guidelines for detailed requirements. View published research articles from PLOS ONE for specific examples.<br><br>This statement is required for submission and will appear in the published article if the submission is accepted. Please make sure it is accurate.<br><br>Funded studies<br>Enter a statement with the following details:<br>• Initials of the authors who received each award<br>• Grant numbers awarded to each author<br>• The full name of each funder<br>• URL of each funder website<br>• Did the sponsors or funders play any role in the study design, data collection and analysis, decision to publish, or preparation of the manuscript?<br><br>Did you receive funding for this work?"	BRAZIL - BR

Powered by Editorial Manager® and ProduXion Manager® from Aries Systems Corporation

Competing Interests<br><br>Use the instructions below to enter a competing interest statement for this submission. On behalf of all authors, disclose any competing interests that could be perceived to bias this work—acknowledging all financial support and any other relevant financial or non-financial competing interests.<br><br>This statement is required for submission and will appear in the published article if the submission is accepted. Please make sure it is accurate and that any funding sources listed in your Funding Information later in the submission form are also declared in your Financial Disclosure statement.<br><br>View published research articles from PLOS ONE for specific examples.<br><br>NO authors have competing interests<br><br>Enter: The authors have declared that no competing interests exist.<br><br>Authors with competing interests<br><br>Enter competing interest details beginning with this statement:<br><br>I have read the journal's policy and the authors of this manuscript have the following competing interests: [insert competing interests here]<br><br>* typeset	NO authors have competing interests
Ethics Statement<br><br>Enter an ethics statement for this submission. This statement is required if the study involved:<br><br>• Human participants<br>• Human specimens or tissue<br>• Vertebrate animals or cephalopods	Human participants. Research Ethics Committee of Universidade Federal de Goiás (protocol number: 4.249.851). The consent was obtained via written and signed TALE and TCLE

Powered by Editorial Manager® and ProduXion Manager® from Aries Systems Corporation

- Vertebrate embryos or tissues
- Field research

Write "N/A" if the submission does not require an ethics statement.

General guidance is provided below. Consult the submission guidelines for detailed instructions. Make sure that all information entered here is included in the Methods section of the manuscript.

Powered by Editorial Manager® and ProduXion Manager® from Aries Systems Corporation

Format for specific study types<br><br>Human Subject Research (involving human participants and/or tissue)<br>• Give the name of the institutional review board or ethics committee that approved the study<br>• Include the approval number and/or a statement indicating approval of this research<br>• Indicate the form of consent obtained (written/oral) or the reason that consent was not obtained (e.g. the data were analyzed anonymously)<br><br>Animal Research (involving vertebrate animals, embryos or tissues)<br>• Provide the name of the Institutional Animal Care and Use Committee (IACUC) or other relevant ethics board that reviewed the study protocol, and indicate whether they approved this research or granted a formal waiver of ethical approval<br>• Include an approval number if one was obtained<br>• If the study involved non-human primates, add additional details about animal welfare and steps taken to ameliorate suffering<br>• If anesthesia, euthanasia, or any kind of animal sacrifice is part of the study, include briefly which substances and/or methods were applied<br><br>Field Research<br><br>Include the following details if this study involves the collection of plant, animal, or other materials from a natural setting:<br>• Field permit number<br>• Name of the institution or relevant body that granted permission	
Data Availability<br><br>Authors are required to make all data underlying the findings described fully available, without restriction, and from the time of publication. PLOS allows rare exceptions to address legal and ethical concerns. See the PLOS Data Policy and FAQ for detailed information.	Yes - all data are fully available without restriction

Powered by Editorial Manager® and ProduXion Manager® from Aries Systems Corporation

A Data Availability Statement describing where the data can be found is required at submission. Your answers to this question constitute the Data Availability Statement and will be published in the article, if accepted.<br><br>Important: Stating 'data available on request from the author' is not sufficient. If your data are only available upon request, select 'No' for the first question and explain your exceptional situation in the text box.<br><br>Do the authors confirm that all data underlying the findings described in their manuscript are fully available without restriction?	
Describe where the data may be found in full sentences. If you are copying our sample text, replace any instances of XXX with the appropriate details.<br><br>• If the data are held or will be held in a public repository, include URLs, accession numbers or DOIs. If this information will only be available after acceptance, indicate this by ticking the box below. For example: All XXX files are available from the XXX database (accession number(s) XXX, XXX.).<br>• If the data are all contained within the manuscript and/or Supporting Information files, enter the following: All relevant data are within the manuscript and its Supporting Information files.<br>• If neither of these applies but you are able to provide details of access elsewhere, with or without limitations, please do so. For example:<br><br>Data cannot be shared publicly because of [XXX]. Data are available from the XXX Institutional Data Access / Ethics Committee (contact via XXX) for researchers who meet the criteria for access to confidential data.<br><br>The data underlying the results presented in the study are available from (include the name of the third party	All relevant data are within the manuscript and its Supporting Information files.

and contact information or URL).<br>• This text is appropriate if the data are owned by a third party and authors do not have permission to share the data.<br><br>* typeset	
Additional data availability information:	Tick here if the URLs/accession numbers/DOIs will be available only after acceptance of the manuscript for publication so that we can ensure their inclusion before publication.

Powered by Editorial Manager® and ProduXion Manager® from Aries Systems Corporation

Manuscript

Click here to access/download;Manuscript;Maintext__VP_03012025.docx

Screening vulnerable populations for hepatitis B, hepatitis C, syphilis, and human immunodeficiency virus during the coronavirus disease 2019 pandemic

Gabriel Francisco da Silva Filho[1¶], Antoninho Barros Milhomem[2&], Bruno Vinícius Diniz e Silva[2&], Kamila Cardoso dos Santos[1¶], Grazielle Rosa da Costa e Silva[1&], Larissa Silva Magalhães[1&], Winny Éveny Alves Moura[1&], Thaynara Lorrane Silva Martins[1&], Wanessa de Oliveira Gonçalves[1&], Roxana Isabel Cardozo Gonzales[1&], Leonora Rezende Pacheco[1&], Regina Maria Bringel Martins[2&], Karlla Antonieta Amorim Caetano[1¶], Megmar Aparecida dos Santos Carneiro[2¶] e Sheila Araújo Teles[1¶*]

[1] Faculty of Nursing, Federal University of Goiás, Goiânia, Goiás, Brazil

[2] Institute of Tropical Medicine and Public Health, Federal University of Goiás, Goiânia, Goiás, Brazil

* Corresponding author

E-mail: sheila.fen@gmail.com (SAT)

[¶]These authors contributed equally to this work.

[&]These authors also contributed equally to this work.

Abstract

During the coronavirus disease 2019 pandemic, we investigated the prevalence of hepatitis B virus (HBV), hepatitis C virus (HCV), syphilis, and human immunodeficiency virus (HIV) among socio-economically vulnerable populations in Goiania, Central Brazil. From August 1st, 2020, to April 28th, 2021, a total of 627 individuals, including lesbian, gay, bisexual, and transgender (LGBT) people, homeless individuals, waste recyclable collectors, and immigrants/refugees, were tested for these infections. Multiple Poisson regression models were used to analyze the factors associated with each outcome. HBV markers were found in 16.1% of participants, antibodies against HCV (anti-HCV) in 1.9%, syphilis in 17.2%, and antibodies against HIV (anti-HIV) in 6.1%. Among these, 32 individuals exhibited serological evidence of active syphilis, whereas HBV deoxyribonucleic acid and HCV ribonucleic acid were detected in six and two individuals, respectively. All affected individuals were referred to health services for appropriate care. Increased age was statistically associated with HCV, HBV, and syphilis; being aware of an HIV diagnosis was linked to HCV and HBV; and identifying as LGBT and reporting a history of sexually transmitted infections was associated with syphilis and HIV. Drug use was associated with HCV and HIV, whereas being an immigrant/refugee or engaging in transactional sex was linked to HBV. Homelessness and reporting a higher number of sexual partners in the previous month were associated with syphilis. Conversely, condom use during the last sexual intercourse served as a protective factor. High prevalence rates of these infections were identified, with many participants showing evidence of active infections, increasing the risk of transmission. Specific risk behaviors were associated with each infection, emphasizing the need to tailor prevention strategies to address these behaviors effectively.

Introduction

Human immunodeficiency virus (HIV), viral hepatitis B and C (HBV and HBC), and sexually transmitted infections (STIs) represent significant global public health concerns and are key targets of the Global Health Sector Strategies 2022–2030. An estimated 3.5 million new cases of HIV, HBV, and HCV are reported annually, whereas one million STIs are diagnosed each day. These infections cause high morbidity and mortality, collectively responsible for 2.5 million deaths and 1.2 million cases each year [1].

HIV causes acquired immunodeficiency syndrome (AIDS) [2]; however, early treatment can prevent AIDS progression [3]. HBV and HCV are significant causes of cirrhosis and hepatocellular carcinoma [4]. A safe and effective HBV vaccine has been available since the 1980s [5], and direct-acting antiviral (DAA) treatments now offer a cure for hepatitis C [6]. Although syphilis can be treated with low-cost antibiotics, the infection remains a cause of neurological and cardiovascular complications in individuals infected with *T. pallidum* [7]. These pathogens share common modes of transmission and prevention measures [1].

Available vaccines, curative treatments, and preventive measures create opportunities to eliminate HIV, viral hepatitis, and syphilis, aligning with the World Health Organization's (WHO) goal to reduce these infections as public health threats by 2030. However, considerable efforts should be made to diagnose infected people and link them to health services for treatment and prevention [8].

In 2023, an estimated 86% of individuals living with HIV knew their serostatus, and 89% of all people living with HIV were receiving antiretroviral therapy. Conversely, by the end of 2022, only 13.4% of the people living with HBV and 36.4% of those with

HCV were aware of their diagnoses, with 2.6% and 20% of diagnosed individuals receiving treatment, respectively [8]. Notably, most individuals living with these infections experience social stigma, inequities, and limited access to health services, which hinders early diagnosis and treatment [8].

The coronavirus disease 2019 (COVID-19) pandemic disproportionally affected economically vulnerable people, and its peak periods disrupted progress toward the control and prevention of STIs [8]. Therefore, during a health crisis, any opportunity to screen for HIV, viral hepatitis, or STI should be considered.

In Brazil, during the first and second waves of the COVID-19 pandemic, our research utilized COVID-19 testing initiatives to screen vulnerable people for HIV, viral hepatitis, and STIs, as recommended by WHO (WHO, 2021b). Therefore, this study shows the prevalence and factors associated with HIV, syphilis, HBV, and HCV in vulnerable populations during the first and second waves of COVID-19 in Goiânia, Central Brazil.

Materials and Methods

Study design, location, population, and sample

This cross-sectional study was conducted in Goiânia, located in Central Brazil, with a population of 1,437,237 inhabitants. The study focused on socially and economically vulnerable groups, including lesbian, gay, bisexual, and transgender (LGBT) people, immigrants/refugees, homeless people, and recyclable waste collectors.

Individuals aged 14 years or older who were assisted by non-governmental organizations (NGOs) catering to the needs of LGBT people, immigrants and refugees, recyclable waste collectors, and homeless individuals were included in the study. Exclusion criteria comprised individuals who appeared to be under the influence of alcohol or illicit drugs during the recruitment process.

The prevalence of HCV previously reported among recyclable waste collectors was considered for sample calculation [8]. Therefore, the required sample size for the study, considering a 95% significance level, a precision of 1.0%, and an anti-HCV prevalence of 1.6% [9], was 605 individuals.

Data collection

In Brazil, at the beginning of the pandemic, COVID-19 testing by public health services was limited for the general population, particularly vulnerable people. Therefore, the Federal University of Goiás offered free COVID-19 testing for socially and economically vulnerable individuals assisted by NGOs. We took advantage of this window of opportunity and invited them to participate in the study on HIV, syphilis, and viral hepatitis B and C. Data collection occurred from August 1st, 2020, to April 28th, 2021, on the University dependencies following biosafety practices. All eligible individuals who agreed to participate signed a written informed consent (IC). Their parents or legal guardians authorized the participation of individuals aged less than 18 years, and a particular written IC was read and signed. In the case of illiteracy, the IC was read to the participant and witness, and the signature was performed by dactyloscopy.

Face-to-face interviews were conducted using a previously validated instrument on sociodemographic and risk factors for STIs. For immigrants and

refugees who did not speak or understand Portuguese, the IC and instrument were translated into French, Haitian Creole, and Spanish, and interviews were conducted by duly trained translators in Creole, French, or Spanish.

After completing the interviews, blood samples (10 mL) were collected from participants to detect the markers of HIV, HBV, HCV, and syphilis. Initially, rapid diagnostic tests were employed, following the manufacturer's instruction, to detect hepatitis B surface antigen (HBsAg) using the HBsAg Bioclin test (Quibasa Química Básica, Belo Horizonte, MG), antibodies to HCV (anti-HCV) using the HCV ab ECO test (Biosensor, ECO Diagnóstica, Corinto, MG), and syphilis (ABONTM Syphilis Ultra Rapid Test, Abbott). For anti-HIV types 1 and 2, initial screening was performed using the ABONTM^{TM} HIV 1/2/O Tri-Line Human Immunodeficiency Virus Rapid Test (Abbott), with reactive samples confirmed using the TR DPP HIV 1/2 (Bio-Manguinhos).

All samples underwent additional testing via an enzyme-linked immunosorbent assay (ELISA) for HBsAg (BIOELISA HBsAg, Bioclin, Quibasa Química Básica, Belo Horizonte, MG), total antibodies to hepatitis B core antigen (anti-HBc total) (Elisa anti-HBc, Wieber Lab., Rosario, Argentina), antibodies to hepatitis B surface antigen (anti-HBs) (BIOELISA Anti-HBs, Bioclin, Quibasa Química Básica, Belo Horizonte, MG), and anti-HCV (BIOELISA HCV, Bioclin, Quibasa Química Básica, Belo Horizonte, MG). Samples reactive for syphilis were further analyzed using the venereal disease research laboratory (VDRL) test (RPR Brás, Laborclin, Pinhais, Paraná). Active syphilis was diagnosed in individuals with VDRL titers ≥ 1/8 in the non-treponemal test.

Samples positive for HBsAg and/or anti-HBc total were subjected to deoxyribonucleic acid (DNA) extraction using the QIAamp® Blood Mini Kit (Qiagen). The pre-S/S region of the HBV genome was amplified through a semi-nested

polymerase chain reaction (PCR) assay with a detection limit of three copies of HBV DNA per reaction, as previously described [10, 11].

Samples positive for anti-HCV were submitted to HCV ribonucleic acid (RNA) detection using reverse transcription (RT) nested PCR with primers targeting the conserved area of the 5' non-coding region of the HCV genome, as outlined in previous studies [12].

Data analysis

The data were collected, entered into EpiData version 3.1, and exported to STATA version 17 SE. Descriptive statistics, including absolute and relative frequencies and measures of central tendency, were used. Parametric and non-parametric tests were used to analyze the differences between means, medians, and proportions, as appropriate. The Poisson multiple regression model included variables with $p < 0.20$. Statistical significance was set at $P < 0.05$.

This study was approved by the Research Ethics Committee of Universidade Federal de Goiás (protocol number: 4.249.851).

Results

A total of 681 individuals participated in the study, of whom 627 were eligible and agreed to blood collection. The participants included 283 recyclable waste collectors, 165 immigrants/refugees, 140 homeless people, and 99 LGBT people. Some participants were categorized into more than one group. The majority were male (53%), mixed race (52.8%), or single race (58.4%). The median age was 34 years (interquartile range [IQR]: 20), the median monthly income was 1,200 BRL (IQR: 976),

and the median years of education were 10 (IQR: 6). Additionally, 14.5% of the participants tested positive for COVID-19 (Table 1).

Table 1. Characteristics of the 627 vulnerable people in Goiânia, Central Brazil.

Category variables	n	%
**Vulnerable population**		
Waste recycle pickers	283	45.1
Immigrants/refugees	165	26.3
Homeless people	140	22.3
LGBT	99	15.8
**Sex**		
Male	332	53.0
Female	295	47.0
**Race/ethnicity**		
White	111	17.7
Black	146	23.3
Mixed	331	52.8
Asian/indigenous	39	6.2
**Marital status**		
Single	362	58.4
Married	212	34.2
Widow/divorced/separated	46	7.4
No information; 7		
**COVID-19**		
No	517	82.5
Yes	91	14.5
Indeterminate	15	2.4
**Aware of HIV diagnosis**		
No	599	95.5

Yes	28	4.5
**Report of physical violence**		
No	596	95.1
Yes	31	4.9
**Report of coercive sex**		
No	625	99.7
Yes	2	0.3
**Anal sex**		
No	366	60.6
Yes	238	39.4
No information; 23		
**Condom use in the last sexual intercourse**		
Yes	238	40.1
No	355	59.9
No information; 34		
**STI report**		
No	495	80.6
Yes	119	19.4
No information; 13		
**Transactional sex**		
No	547	89.7
Yes	63	10.3
No information; 17		
**Illicit drug use**		
No	424	68.1

Yes	199	31.9
No information; 4		
**Daily alcohol consumption**		
No	598	95.4
Yes	29	4.6
**Previous arrest**		
No	517	84.1
Yes	98	15.9
No information; 12		
**Continuous variables**	**Median**	**IQR[a]**
Age	34	20
Monthly income (R$)	1,200	976
Years of education	10	6.0
Number of sexual partners (last month)	1	0

Legend:

* IQR: interquartile range

[a] STI: sexually transmitted infection

Of the total participants, 31 (4.9%) reported a history of physical violence, and two (0.3%) reported coercive sex. A history of STIs was found in 19.4% of participants, and 10.3% reported engaging in transactional sex. Additionally, 39.4% of participants reported engaging in anal sex, and 59.9% did not use condoms during their last sexual intercourse. Nearly one-third of the participants (31.9%) reported a history of drug use. Daily alcohol consumption was reported by 4.6% of the participants, and 15.9% had been arrested. Moreover, 4.5% of participants were aware of their HIV diagnosis.

1

1

HBV exposure markers (HBsAg and anti-HBc) were found in 101 (16.1%; 95% confidence interval [CI]: 13.4–19.2%) individuals, anti-HCV in 1.9% (95% CI: 1.1–3.3%), anti-HIV in 6.1% (95% CI: 4.4–8.2%), and syphilis in 17.2% (95% CI:14.5–20.4%). Among 108 individuals who tested positive for the treponemal test, 32 (29.63%) also tested positive for the VDRL test (≥ 1/8) (Table 2). Additionally, HBV DNA was detected in six out of 101 anti-HBc positive samples: three anti-HBc positive/HBsAg positive and three anti-HBc positive/HBsAg negative (occult HBV infection). HCV RNA was detected in two out of 12 anti-HCV positive samples.

Table 2. Prevalence of HBV, HCV, syphilis, and HIV serological markers among vulnerable people in Goiânia, Central Brazil.

Markers	Pos./total	(%)	95% CI
**HBV**			
**HBsAg+**	2/627	0.32	0.09–1.16
**HBsAg+Anti-HBc+**	5/627	0.80	0.34–1.85
**Anti-HBc only+**	20/627	3.19	2.07–4.86
**Anti-HBc+/anti-HBs+**	74/627	11.96	9.65–14.74
**Any exposure marker**	101/627	16.11	13.44–19.19
**Anti-HBs (immunized)+**	250/627	39.87	36.11–43.76
**HCV**			
**Anti-HCV+**	12/627	1.91	1.10–3.32
**HIV**			
**Anti-HIV+**	38/627	6.1	4.45–8.21
**Syphilis**			
**Anti-*T. Pallidum*+**	108/627	17.2	14.47–20.38
**VDRL ≥ 1:8+**	32/108	29.63	21.84–38.82

In the bivariate analysis, considering the HCV outcome, the variables, including homelessness, age, schooling, monthly income, number of sexual partners (lifetime), anal sex, condom use, STI report, drug use, previous arrest, and awareness of HIV diagnosis showed a *p*-value < 0.20 (S1 Table) and were included in a multiple regression model. The final model showed that for each year of age, the prevalence of anti-HCV increased by 8.5% (adjusted prevalence ratio [APR]: 1.085; 95% CI: 1.041–1.130). Individuals who used illicit drugs presented an APR of 5.858 (95% CI: 1.212–28.312) compared to non-drug users. Moreover, individuals who were aware of their HIV diagnosis showed an APR of 3.673 (95% CI: 1.391–9.695) than their counterparts (Table 3).

Table 3. Variables associated with HBV, HCV, Syphilis, and HIV among vulnerable people in Goiânia, Brazil Central.

**HCV[a]**	**p-value**	**APR (95% CI)**
Homeless	0.090	2.565 (0.862–7.632)
Age	< 0.001	1.085 (1.041–1.130)
Illicit drug use	< 0.028	5.858 (1.212–28.312)
Aware of HIV diagnosis	< 0.009	3.673 (1.391–9.695)
**HBV[b]**	**p-value**	**APR (95% CI)***
Immigrants/refugees	< 0.001	2.582 (1.791–3.724)
Age	< 0.001	1.023 (1.011–1.035)
Aware of HIV diagnosis	< 0.001	2.204 (1.452–3.344)
Transactional sex	0.001	1.907 (1.291–2.816)
**Syphilis[c]**	**p-value**	**APR (95% CI)**
Homeless	0.001	1.866 (1.304–2.670)
LGBT	< 0.001	1.919 (1.349–2.731)
Age	0.043	1.012 (1.001–1.025)
Number of sexual partners in the last month	0.023	1.001 (1.000–1.002)

STI report	< 0.001	3.371 (2.280–4.985)
Condom use in the last sexual intercourse	0.032	0.683 (0.482–0.967)
**HIV[d]**	**p-value**	**APR (95% CI)**
Waste recycle pickers	0.057	0.201 (0.038–1.050)
Homeless people	0.274	1.390 (0.770–2.508)
LGBT	0.032	2.209 (1.072–4.553)
STI report	< 0.001	12.076 (4.577–31.859)
Illicit drug use	0.036	1.889 (1.041–3.452)
Condom use in the last sexual intercourse	0.055	0.535 (0.283–1.041)

Legend:

*APR (95% CI); adjusted prevalence ratio; 95% confidence interval.

[a]Adjusted by homeless, age, years of education, monthly income, aware of HIV diagnosis, condom use, anal sex, IST, previous arrest, drug use; [b]Adjusted by immigrant/refugee, waste recycler picker, LGBT, age, STI, aware of HIV diagnosis, condom use, transactional sex, drug use; [c]Adjusted by homeless, LGBT, immigrant/refugee, waste recycler picker, age, anal sex, physical violence, condom use, drug use, number of sexual partners, aware of HIV diagnosis, previous arrest, age, and alcohol daily consumption; [d]Adjusted by homeless, LGBT, waste recycle picker, immigrant/refugee, gender, physical violence, drug use, IST, condom use, and transactional sex.

HBV exposure was significantly associated with being waste recycling pickers, immigrants/refugees, age, STI reports, and awareness of one's own HIV diagnosis ($p < 0.05$) (S2 Table). These variables, along with being LGBT, number of sexual partners (lifetime), non-condom use, transactional sex, and drug use were included in a multiple Poisson regression model, and being immigrants/refugees (APR: 2.582; 95% CI: 1.179–3.724), aging (APR: 1.023; 95% CI: 1.011–1.035); awareness of own HIV

diagnosis (APR: 2.204; 95% CI: 1.452–3.344) and transactional sex (APR: 1.907; 95% CI: 1.291–2.816) remained independently associated with HBV markers (Table 3).

Initially, syphilis was associated with all vulnerable groups studied plus age, physical violence, number of sexual partners (lifetime), anal sex, non-condom, STI report, transactional sex, non-injection drug use, injection drug use, daily alcohol consumption, previous arrest, and awareness of HIV diagnosis (S3 Table). Multiple regression analysis included these variables and the number of sexual partners in the last month (*p*=0.150). The variables associated with syphilis in the final model were: being homeless people (RPA: 1.866; 95% CI: 1.304–2.670), being LGBT (RPA: 1.919; 95% CI: 1.349–2.731), aging (RPA:1.012; 95% CI: 1.001–1.025), increased number of sexual partners in the last month (RPA: 1.001; 95% CI: 1.000–1.002), and report of STI 3.371 (95% CI: 2.280–4.985), whereas condom use in the previous sexual intercourse was a protective factor (RPA: 0.683; 95% CI: 0.482–0.967) (Table 3).

All vulnerable groups were also associated with HIV in bivariate analysis, besides anal sex, non-condom use, STI report, transactional sex, drug use (non-injection and injection), and previous arrest (S4 Table). The multiple regression analysis included these variables and sex, number of sexual partners (lifetime), and physical violence. The final model showed that LGBT people (RPA: 2.209; 95% CI: 1.072–4.553), STI report (RPA: 12.076; 95% CI: 4.577–31.859), and illicit drug use (RPA: 1.889; 95% CI: 1.041–3.452) remained associated with HIV (Table 3).

Discussion

The COVID-19 pandemic represented the worst health crisis of the 21st century. Besides the direct effects of the novel coronavirus, which has caused elevated morbidity and mortality globally, several health programs have been compromised. Therefore, optimized efforts are required to overcome this health sector crisis [13]. We took advantage of COVID-19 screening and tested vulnerable individuals for HBV, HCV, syphilis, and HIV. Additionally, active infections were identified and referred to by public health services.

In this study, HIV prevalence among the vulnerable groups reached 6.1%, a rate 15 times higher than the prevalence in the Brazilian general population (0.4%) [14]. Syphilis prevalence (17.2%) significantly exceeded that of Brazilian sentinel populations, such as male conscripts (1.09%) [15] and pregnant women (1.02%) [16]. The anti-HCV prevalence was 1.91%, almost four times greater than the general population estimate of 0.53% [17]. HBV exposure markers were detected in 16.1% of participants, slightly higher than the national prevalence of 11.6%, likely reflecting the protective effect of the hepatitis B vaccine, which has been universally available in Brazil [18]. These findings underscore the disproportionate burden of STIs among vulnerable people and the necessity of creating opportunities for regular screening to meet the global goal of eliminating these infections by 2030. Moreover, the significant proportion of individuals with active infections and high-risk sexual behaviors emphasizes their potential role in further transmission, reinforcing the need for targeted public health interventions.

Statistical analysis revealed common variables associated with the outcomes. The association between aging and HCV, HBV, and syphilis reflects the cumulative opportunities for exposure to these agents throughout life. Being aware of an HIV diagnosis was associated with HCV and HBV infections. Viral hepatitis B and C and

HIV coinfections are common. Two systematic reviews and meta-analyses estimated the global prevalence of HBV and HCV among people living with HIV. The prevalence of HCV/HIV ranges from 2.4% in the general population to 82.4% among individuals who inject drugs [19]. For HBV, HBsAg prevalence ranges from 6.1% in heterosexual/pregnant and men who have sex with men to 11.8% in people who inject drugs [20]. Individuals co-infected with HIV and viral hepatitis B and/or C are at a higher risk of accelerated progression of liver diseases and HCC [21].

LGBT people were associated with syphilis and HIV, highlighting the importance of driving preventive measures for this key population [1]. STI report was also associated with syphilis and HIV, representing a surrogate marker of unsafe sex and a sensitive question for STI screening.

Psychoactive drugs increase sexual desire, arousal, and risk-taking behavior [22]. Therefore, a high frequency of HIV and HCV has been observed among illicit drug users [23, 24]. A strong association was detected between drug use and HCV and HIV. In countries where HCV screening in blood banks includes nucleic acid tests (NAT) and safety care procedures, drug injection use represents the major risk factor for HCV [25, 26]. Therefore, offering HCV testing for injection drug users and linking them to health services for DAA treatment is imperative to eliminate HCV as a global public health problem [1]. In Brazil, these medicines are free of charge for all populations [27], and the challenge is to reach people who live mostly outside the reach of public health services.

Brazil has become a destination for immigrants and refugees in recent decades, mainly from Latin American countries [28]. This study identified a 2.6-fold higher APR for HBV exposure among immigrants and refugees compared to other

vulnerable groups, highlighting the importance of hepatitis B vaccination and testing for these emergent population groups. HBV is efficiently transmitted sexually in countries with low endemicity, making sexual practices a significant focus for preventive measures [29].

An increased number of sexual partners in the previous month was associated with syphilis infection. This, combined with low condom use during sexual activities, represents the cornerstone of STI dissemination. Surprisingly, not using a condom during the last sexual intercourse was a protective factor against syphilis. However, this result should be considered cautiously, and reverse causality should not be ruled out. Individuals with syphilis may be more conscious of safe sex.

Homeless people were significantly associated with syphilis, consistent with previous studies showing a high prevalence in this population [30, 31]. A previous investigation in Goiânia reported a lifetime syphilis prevalence of 22%, confirming their elevated risk for this infection [32].

This study had limitations. Firstly, the cross-sectional design limited the ability to establish causality between risk factors and infections. Secondly, the use of a convenience sample may not have fully represented the broader population of vulnerable individuals. Despite these limitations, the findings were consistent with those reported in the existing literature.

During the COVID-19 pandemic, this study successfully tested vulnerable populations for STIs, revealing a high prevalence of the investigated infections and identifying multiple risk factors associated with their transmission. All individuals testing positive were referred to public health services specializing in STIs. Overcoming

barriers in the health sector during crises remains crucial to effectively address the healthcare needs of at-risk populations.

Acknowledgments

We express our sincere gratitude to all collaborators whose contributions were vital to this study. We deeply appreciate the participants who generously shared their time and experiences, making this research possible. We are also thankful to the institutions and facilities that kindly provided their spaces and resources, enabling the execution of experiments and sample collection. Your support and commitment have been invaluable to the success of this work.

References

1. WHO. Global health sector strategies on, respectively, HIV, viral hepatitis and sexually traansmitted infections for the period 2022-2030. Geneva: World Health Organization; 2022. p. 126.
2. Montagnier L. 25 years after HIV discovery: prospects for cure and vaccine. Virology. 2010;397(2):248-54. Epub 2010/02/16. doi: 10.1016/j.virol.2009.10.045. PubMed PMID: 20152474.
3. WHO. Consolidated guidelines on HIV prevention, testing, treatment, servicedelivery and monitoring: recommendations for a public health approach. Geneva: World Health Organization; 2021. p. 592.
4. Lanini S, Ustianowski A, Pisapia R, Zumla A, Ippolito G. Viral Hepatitis: Etiology, Epidemiology, Transmission, Diagnostics, Treatment, and Prevention. Infect Dis Clin North Am. 2019;33(4):1045-62. Epub 2019/11/02. doi: 10.1016/j.idc.2019.08.004. PubMed PMID: 31668190.

5. Krugman S. Hepatitis virus vaccines: present status. Yale J Biol Med. 1982;55(3-4):375-81. Epub 1982/05/01. PubMed PMID: 6295013; PubMed Central PMCID: PMCPMC2596435.
6. Ghany MG, Morgan TR, AASLD-IDSA Hepatitis C Guidance Panel. Hepatitis C Guidance 2019 Update: American Association for the Study of Liver Diseases-Infectious Diseases Society of America Recommendations for Testing, Managing, and Treating Hepatitis C Virus Infection. Hepatology. 2020;71: 686–721. doi:10.1002/hep.31060.
7. Ghanem KG, Ram S, Rice PA. The Modern Epidemic of Syphilis. N Engl J Med. 2020;382(9):845-54. Epub 2020/02/27. doi: 10.1056/NEJMra1901593. PubMed PMID: 32101666.
8. WHO. Global progress report on HIV, viral hepatitis and sexually transmitted infections, 2021. World Health Organization; 2021. p. 112.
9. Marinho TA, Lopes CL, Teles SA, Reis NR, Carneiro MA, de Andrade AA, et al. Prevalence of hepatitis C virus infection among recyclable waste collectors in Central-West Brazil. Mem Inst Oswaldo Cruz. 2013;108(4):519-22. Epub 2013/07/06. doi: 10.1590/S0074-02762013000400021. PubMed PMID: 23828009; PubMed Central PMCID: PMCPMC3970612.
10. Niel C, Moraes MT, Gaspar AM, Yoshida CF, Gomes SA. Genetic diversity of hepatitis B virus strains isolated in Rio de Janeiro, Brazil. J Med Virol. 1994;44(2):180-6. PubMed PMID: 7852959.
11. Motta-Castro AR, Martins RM, Araujo NM, Niel C, Facholi GB, Lago BV, et al. Molecular epidemiology of hepatitis B virus in an isolated Afro-Brazilian

community. Arch Virol. 2008;153(12):2197-205. doi: 10.1007/s00705-008-0237-0. PubMed PMID: 18998047.

12. Ginabreda MG, Yoshida CF, Niel C. Genomic characterization of Brazilian hepatitis C virus genotypes 1a and 1b. Braz J Med Biol Res. 1997;30(3):339-45. PubMed PMID: 9246231.

13. WHO. Global progress report on HIV, viral hepatitis and sexually transmitted infections, 2021. . Accountability for the global health sector strategies 2016–2021: actions for impact. Geneva: World Health Organization; 2021. p. 112.

14. Brasil. Boletim Epidemiológico de AIDS e DST. Brasília: Ministério da Saúde, 2014 1517-1159.

15. Motta LR, Sperhacke RD, Adami AG, Kato SK, Vanni AC, Paganella MP, et al. Syphilis prevalence and risk factors among young men presenting to the Brazilian Army in 2016: Results from a national survey. Medicine (Baltimore). 2018;97(47):e13309. Epub 2018/11/22. doi: 10.1097/MD.0000000000013309. PubMed PMID: 30461642; PubMed Central PMCID: PMCPMC6393142.

16. Domingues RM, Szwarcwald CL, Souza Junior PR, Leal Mdo C. Prevalence of syphilis in pregnancy and prenatal syphilis testing in Brazil: birth in Brazil study. Rev Saude Publica. 2014;48(5):766-74. Epub 2014/11/06. doi: 10.1590/s0034-8910.2014048005114. PubMed PMID: 25372167; PubMed Central PMCID: PMCPMC4211581.

17. Benzaken AS, Girade R, Catapan E, Pereira GFM, Almeida EC, Vivaldini S, et al. Hepatitis C disease burden and strategies for elimination by 2030 in Brazil. A mathematical modeling approach. Braz J Infect Dis. 2019;23(3):182-90. Epub

2019/05/31. doi: 10.1016/j.bjid.2019.04.010. PubMed PMID: 31145876; PubMed Central PMCID: PMCPMC9428180.

18. Souto FJD. Distribution of hepatitis B infection in Brazil: the epidemiological situation at the beginning of the 21st century. Revista da Sociedade Brasileira de Medicina Tropical. 2016;49(1):11-23.

19. Platt L, Easterbrook P, Gower E, McDonald B, Sabin K, McGowan C, et al. Prevalence and burden of HCV co-infection in people living with HIV: a global systematic review and meta-analysis. Lancet Infect Dis. 2016;16(7):797-808. Epub 2016/02/29. doi: 10.1016/S1473-3099(15)00485-5. PubMed PMID: 26922272.

20. Platt L, French CE, McGowan CR, Sabin K, Gower E, Trickey A, et al. Prevalence and burden of HBV co-infection among people living with HIV: A global systematic review and meta-analysis. J Viral Hepat. 2020;27(3):294-315. Epub 2019/10/12. doi: 10.1111/jvh.13217. PubMed PMID: 31603999; PubMed Central PMCID: PMCPMC7383613.

21. Hu J, Liu K, Luo J. HIV-HBV and HIV-HCV Coinfection and Liver Cancer Development. Cancer Treat Res. 2019;177:231-50. Epub 2018/12/14. doi: 10.1007/978-3-030-03502-0_9. PubMed PMID: 30523627.

22. Frohmader KS, Pitchers KK, Balfour ME, Coolen LM. Mixing pleasures: review of the effects of drugs on sex behavior in humans and animal models. Horm Behav. 2010;58(1):149-62. Epub 2009/12/17. doi: 10.1016/j.yhbeh.2009.11.009. PubMed PMID: 20004662.

23. Luo T, Lin Z, Wu Z, Cen P, Nong A, Huang R, et al. Trends and associated factors of HIV, HCV and syphilis infection among different drug users in the

China-Vietnam border area: an 11-year cross-sectional study (2010-2020). BMC Infect Dis. 2023;23(1):575. Epub 2023/09/05. doi: 10.1186/s12879-023-08239-3. PubMed PMID: 37667212; PubMed Central PMCID: PMCPMC10478360.

24. Aghaei AM, Gholami J, Sangchooli A, Rostam-Abadi Y, Olamazadeh S, Ardeshir M, et al. Prevalence of injecting drug use and HIV, hepatitis B, and hepatitis C in people who inject drugs in the Eastern Mediterranean region: a systematic review and meta-analysis. Lancet Glob Health. 2023;11(8):e1225-e37. Epub 2023/07/21. doi: 10.1016/S2214-109X(23)00267-X. PubMed PMID: 37474230.

25. Degenhardt L, Charlson F, Stanaway J, Larney S, Alexander LT, Hickman M, et al. Estimating the burden of disease attributable to injecting drug use as a risk factor for HIV, hepatitis C, and hepatitis B: findings from the Global Burden of Disease Study 2013. Lancet Infect Dis. 2016;16(12):1385-98. Epub 2016/09/26. doi: 10.1016/S1473-3099(16)30325-5. PubMed PMID: 27665254.

26. Spearman CW, Dusheiko GM, Hellard M, Sonderup M. Hepatitis C. Lancet. 2019;394(10207):1451-66. Epub 2019/10/22. doi: 10.1016/S0140-6736(19)32320-7. PubMed PMID: 31631857.

27. Brasil. Protocolo Clínico e Diretrizes Terapêuticas para Hepatite C e Coinfecções In: Secretaria de Vigilância em Saúde. Departamento de Vigilância PeCdIST, do HIV/Aids e das Hepatites Virais, editor. Brasilia: Ministério da Saúde; 2019. p. 68.

28. Cavalcanti L, Oliveira T, Silva BG. 2011-2020: Uma década de desafios para a imigração e refúgio no Brasil. In: Migrações, editor. Brasilia: Ministério da

Justiça e Segurança Pública/ Conselho Nacional de Imigração e Coordenação Geral de Imigração Laboral.; 2021. p. 321.

29. Odenwald MA, Paul S. Viral hepatitis: Past, present, and future. World J Gastroenterol. 2022;28(14):1405-29. Epub 2022/05/19. doi: 10.3748/wjg.v28.i14.1405. PubMed PMID: 35582678; PubMed Central PMCID: PMCPMC9048475.

30. Felipetto LG, Teider-Junior PI, da Silva FFV, Couto ACD, Kmetiuk LB, Martins CM, et al. Serosurvey of anti-treponema pallidum (syphilis), anti-hepatitis C virus and anti-HIV antibodies in homeless persons of Sao Paulo city, southeastern Brazil. Braz J Infect Dis. 2021;25(4):101602. Epub 2021/08/15. doi: 10.1016/j.bjid.2021.101602. PubMed PMID: 34389284; PubMed Central PMCID: PMCPMC9392160.

31. Ly TDA, Hoang VT, Louni M, Dao TL, Badiaga S, Tissot-Dupont H, et al. Epidemiological serosurvey and molecular characterization of sexually transmitted infections among 1890 sheltered homeless people in Marseille: Cross-sectional one day-surveys (2000-2015). J Infect. 2021;82(1):60-6. Epub 2020/12/07. doi: 10.1016/j.jinf.2020.11.026. PubMed PMID: 33279534.

32. Pinheiro RS, Carvalho P, Matos MA, Caetano KAA, Paula AC, Carneiro M, et al. Human immunodeficiency virus infection and syphilis among homeless people in a large city of Central-Western Brazil: prevalence, risk factors, human immunodeficiency virus-1 genetic diversity, and drug resistance mutations. Braz J Infect Dis. 2021;25(1):101036. Epub 2020/11/29. doi: 10.1016/j.bjid.2020.11.001. PubMed PMID: 33248020; PubMed Central PMCID: PMCPMC9392128.

Supporting information

S1 Table. Bivariate analysis of potential variables associated with anti-HCV among vulnerable people in Goiânia, Central Brazil.

S2 Table. Bivariate analysis of potential variables associated with HBV among vulnerable people in Goiânia, Central Brazil.

S3 Table. Bivariate analysis of potential variables associated with syphilis among vulnerable people in Goiânia, Central Brazil.

S4 Table. Bivariate analysis of potential variables associated with HIV among vulnerable people in Goiânia, Central Brazil.

S1_table

Click here to access/download
Supporting Information
S1_table.docx

S2_table

Click here to access/download
Supporting Information
S2_Table.docx

S3_table

Click here to access/download
Supporting Information
S3_Table.docx

S4_table

Click here to access/download
Supporting Information
S4_Table.docx
