# Supplementary material for: Taking the opportunity of COVID testing to screen vulnerable populations for hepatitis B, hepatitis C, syphilis, and human immunodeficiency virus in Central Brazil
Source: PLoS One. 2025 Jul 11;20(7):e0325859. doi: 10.1371/journal.pone.0325859 (PMC12250198; doi:10.1371/journal.pone.0325859)
Supplement: Attachment — Submitted filename: R_Response_to_reviewers.docx [file pone.0325859.s007.docx]

March 3rd, 2025

Pierre Roques, Ph.D.

Academic Editor

PLOS ONE

Dear Editor,

Thank you for your careful consideration. We appreciate all the suggestions from your reviewers about our manuscript "Screening vulnerable populations for hepatitis B, hepatitis C, syphilis, and human immunodeficiency virus during the coronavirus disease 2019 pandemic (PONE-D-24-55277)". We agree with the reviewer’s comments and carefully attempted to address their questions and comments.

**Reviewer #1:** The study is interesting, but I have some reservations and suggestions that would potentially improve the manuscript.

**Q1a** *Overall much is said about the covid 19 context but in fact, although the study used the covid 19 context to do the study I think it has nothing to do with covid. Unless the authors hypothesize that the prevalences and risk factors are linked to covid it should be explicit that the study just piggybacked the covid intervention...If the authors do believe that covid is a central part of their hypothesis they should be explicit and state how the design addresses that hypothesis.*

R1a: We appreciate the reviewer's comment and understand the need to clarify the role of COVID-19 in our study. Our research does not hypothesize that the prevalence and risk factors of HIV, viral hepatitis, and STIs are directly linked to the COVID-19 pandemic. Instead, we leveraged the COVID-19 testing initiatives as an opportunity to screen vulnerable populations for these infections, as recommended by the WHO. To address this concern, we have revised the introduction to clarify that the study was conducted during the pandemic context to access vulnerable populations instead of examining a direct relationship between COVID-19 and the infections studied. The revised paragraph now reads in lines 76-86:

*"*The coronavirus disease 2019 (COVID-19) pandemic disproportionally affected economically vulnerable populations, and its peak periods disrupted progress in controlling and preventing STIs. Therefore, WHO recommended taking advantage of COVID-19 testing initiatives to screen vulnerable people for HIV, viral hepatitis, and STIs [8].”

**Q1b** *Why were persons using alcohol or drugs excluded? One could argue that they are a vulnerable group. The population studied seems like a composite group that is "vulnerable" but quite heterogenous Waste collectors, homeless and lgbt are very different worlds and sexual behaviors. There seems to be an opportunistic aspect to this choice, to have a bigger sample size*

R1b: As described in lines 90 and 91, we excluded only individuals under psychoactive substances during the data collection because those individuals could not respond appropriately to the research questions. A hundred ninety-nine individuals reported they used illicit drugs, and 29 used alcohol (Table 1).

Concerning the group studied, as described in Methods, during the first waves of the pandemic, our university offered COVID testing free of charge for socially and economically vulnerable individuals assisted by NGO partners. Although we agree they are not homogeneous (considering some risky behaviors), they share health determinants such as low income, low education, and historically low access to health services. In addition, there are overlaps between groups.

**Q1c** *For the models the risk is not necessarily linearly associated with age so I suggest using age categories to compute APR.*

R1c: Following your suggestion, we compared the models, including age as a dummy variable (≤ 35 years;> 35 years, based on median age) vs. age as a continuous variable, and the models fit better with age as a continuous variable. Therefore, we ask you to consider the current models.

**Q1d** *In the results the text repeats values and 95%CI that are already in the table. I think it is a bit redundant.*

R1d: Thank you for your careful review. We have revised the results section to remove unnecessary repetitions while ensuring the key findings remain clearly described in the text.

**Q1e** *Since we are mostly talking about sexual risk there should be a table with the sexual risks per subpopulation (what proportion of waste collectors used condoms, engaged in anal sex, translational sex...etc for refugees and migrants lgbt....)*

Q1e: Thank you for your careful review. We included S1Table, with information about sociodemographic characteristics and sexual risk per group.

**Reviewer #2:** Dear authors, I have read the article entitled “Screening vulnerable populations for hepatitis B, hepatitis C, syphilis, and human immunodeficiency virus during the coronavirus disease 2019 pandemic”. I have some minor concerns:

**Q2a** For better understanding, the data description in each table should be given before the tables appear (Lines: 172-178; lines: 212-237).

R2a: Thank you for your careful review. We have revised the manuscript to ensure that the data description precedes the corresponding tables in the specified sections.

Q2b *In addition, as each table corresponds to a part of the results, each part must have a title.*

R2b: We have revised the manuscript to ensure that each section of the results corresponds to a clearly defined title, aligning with the respective tables.

**Q2c** *Lines 183-186: These data are not shown in Table 2.*

R 2c: Thank you for your careful review. HBV DNA and HCV RNA detection results were presented in another paragraph. Please let us know if you agree.

**Reviewer #3:**

**Q3a** *Line 1 to 3: The authors could specify the study area in the title*.

R3a: Thank you for your careful review. We considered your suggestion and explicitly modified the title to include the study area (**"**Taking the opportunity of COVID testing to screen vulnerable populations for hepatitis B, hepatitis C, syphilis, and human immunodeficiency virus in Goiás, Central Brazil). Please let us know if you agree with this new version.

**Q3b** *Line 23: The 273-word abstract highlights the important results of this study. However, it would be interesting to highlight the context and objectives of the study at the beginning of the summary.*

R3b: Thank you for your careful review. We have revised the abstract to highlight the context and objectives of the study at the beginning, ensuring a clearer introduction to the research.

**Q3c** *Harmonize this reference with the previous ones which are in the numbered style.*

R3c: Thank you for your careful review. The reference was harmonized as recommended.

**3d** *Line 144 to 147: Which extraction kit was used for HCV RNA extraction?*

R3d: We added the kit’s name to the text. QIAamp® Viral RNA Mini Kit; Qiagen, Hilden, Germany.

**3e** *Line 154: Make the P lowercase. Harmonize with the previous one.*

R3e: Thank you for your careful review. The **p** has been harmonized with the other instances throughout the manuscript to ensure consistency.

**3f** *Line 167 (table 1): The sum of the percentages exceeds 100; there are certainly groups that overlap; could you separate them?*

R3f: Thank you for your careful review. We included the information on overlap groups in the Table’s Legend.

**Q3g** *Conclusion: The manuscript does not clearly highlight the correlation between COVID-19 and other infections. I therefore suggest that we remove the term from the title.*

R3g: Thank you for your careful review. We replaced the title with **"**Taking the opportunity of COVID testing to screen vulnerable populations for hepatitis B, hepatitis C, syphilis, and human immunodeficiency virus in Central Brazil." This new title ensures that the study is accurately represented while avoiding any implication of a causal relationship between COVID-19 and the infections analyzed.
